# Mitochondrial Dysfunction: From Molecular Mechanisms to Modern Approaches for Basic and Clinical Research

**DOI:** 10.3390/biomedicines14071521

**Published:** 2026-07-07

**Authors:** Tatiana V. Kirichenko, Yaroslav D. Tolkachev, Stepan M. Bessonov, Alexander M. Markin, Yuliya V. Markina

**Affiliations:** 1 Petrovsky National Research Centre of Surgery, 119435 Moscow, Russia; t-gorchakova@mail.ru (T.V.K.); alexander.markin.34@gmail.com (A.M.M.); 2 Petrovsky Medical University, 119435 Moscow, Russia

**Keywords:** mitochondrial dysfunction, mtDNA, transcriptome analysis, seahorse assay, flow cytometry, cardiovascular disease, oncology, neurodegenerative diseases, metabolic syndrome

## Abstract

Mitochondria play a vital role in fundamental cellular processes, serving as key regulators of energy metabolism, apoptosis, oxidative stress, calcium homeostasis. Mitochondrial dysfunction is widely regarded as a common pathogenic pathway in the development of widespread chronic diseases, such as metabolic disorders, cardiovascular disease, neurodegeneration, and malignancies. Modern research examines mitochondrial dynamics, mitophagy, mitochondrial biogenesis, mtDNA damage, and the role of reactive oxygen species not only for in-depth understanding of disease pathogenesis but also for identifying diagnostic markers and therapeutic targets. Determining mitochondrial dysfunction is a significant challenge and should involve a comprehensive approach with reliable assessment methods that take into account the dynamic state, number, and localization of mitochondria. The review summarizes the results of the studies exploring the pathogenetic role of mitochondrial dysfunction in the development of widespread chronic diseases and current methods of its evaluation for the integration of mitochondrial dysfunction biomarkers into modern diagnostic strategies and development of mitochondria-target treatment approaches.

## 1. Introduction

Mitochondria play a vital role in fundamental cellular processes, serving as key regulators of energy metabolism, apoptosis, oxidative stress, calcium homeostasis, and more. Mitochondrial dysfunction is widely regarded as a universal pathogenetic mechanism in the development of many socially significant diseases, including metabolic disorders, cardiovascular diseases, neurodegeneration, and oncology [1]. The study of mitochondrial dysfunction is not only fundamental for understanding cellular pathophysiology but also has practical value for identifying early biomarkers, assessing disease severity, and developing new therapeutic approaches [2]. Therefore, interest in mitochondrial dysfunction is steadily growing.

Modern research examines mitochondrial dynamics, mitophagy, mitochondrial biogenesis, mtDNA damage, and the role of reactive oxygen species not only for in-depth understanding of disease pathogenesis but also for identifying diagnostic markers and therapeutic targets [3]. Mitochondrial dysfunction is assessed using various methods, including flow cytometry for measuring membrane potential, the Seahorse assay for assessing mitochondrial respiration parameters in real time, qPCR or NGS sequencing for determining mtDNA heteroplasmy, and others [4,5]. Determining mitochondrial dysfunction is a significant challenge and should involve a comprehensive approach with reliable assessment methods that take into account the dynamic state, number, and localization of mitochondria.

This review summarizes the results of studies examining the pathogenetic role of mitochondria in the development of socially significant diseases, highlighting the relevance of diagnosing mitochondrial dysfunction in clinical and experimental studies. Modern methods for assessing mitochondrial function are critically examined for the integration of mitochondrial dysfunction biomarkers into modern diagnostic strategies and the development of mitochondrial-targeted treatment approaches. The advantages and limitations of diagnostic approaches and individual biomarkers are discussed, along with prospects for their implementation in clinical practice.

This narrative review included manuscripts published in the English language and indexed in the PubMed and Scopus databases up to May 2026, focusing primarily on the last 5 years. The search was performed using combinations of keywords and Boolean operators to capture both the general topic of mitochondrial dysfunction and its association with specific diseases and methodological approaches. In Section 2, we analyzed publications addressing the role of mitochondrial dysfunction in the pathogenesis of cardiovascular diseases (atherosclerosis and cardiomyopathy), neurodegenerative diseases (Parkinson’s, Alzheimer’s, Huntington’s disease and amyotrophic lateral sclerosis), metabolic syndrome (obesity, type 2 diabetes mellitus, and non-alcoholic fatty liver disease) and oncology. To this end, the search strategy incorporated terms related to these diseases. In Section 3, the literature selection focused on experimental and analytical methods for studying mitochondrial dysfunction. The search employed keywords such as mtDNA, qPCR, single-cell sequencing, transcriptome analysis, the Seahorse assay, flow cytometry, and electron microscopy. A full-text analysis of the publications most relevant to the aim of the review ensured a representative set of sources reflecting both the clinical aspects of mitochondrial dysfunction and the current methods used to study it.

## 2. Mitochondrial Dysfunction in the Pathogenesis of Widespread Chronic Diseases

### 2.1. Cardiovascular Diseases

#### 2.1.1. Atherosclerosis

Atherosclerosis is a chronic arterial disease characterized by the formation of atherosclerotic plaques—accumulations of cholesterol and lipids in the inner layer of the artery wall. These plaques impair blood flow and can also cause thromboembolism in the case of rupture. Endothelial dysfunction is a critical predictor of atherosclerosis. The synthesis of nitric oxide (NO) by endothelial nitric oxide synthetase (eNOS) is essential for normal vascular endothelial function and vasodilation. Oxidative stress, which results from an imbalance between reactive oxygen species (ROS) production and the function of the antioxidant system, including superoxide dismutase (SOD), catalase, and glutathione peroxidase (GPx), leads to increased ROS formation, specifically superoxide, at complexes I and III of the electron transport chain in mitochondria. Oxidative stress leads to oxidation of the BH4 cofactor, which causes uncoupling of eNOS, stimulates synthesis of superoxide, which aggravates oxidative stress [6]. Superoxide damages mitochondrial membrane proteins and mitochondrial DNA (mtDNA), which normally activates mitophagy, but during metabolic stress, an accumulation of damaged organelles occurs. It was demonstrated in a model of old mice aged 18–19 months that induced atherosclerosis is associated with increased level of the mitophagy activator Parkin more than 2-fold, polyubiquitination of mitochondria, disruption of oxidative phosphorylation, decreased maximum respiration of mitochondria by 40–50% and ROS production [7]. Oxidative stress activates the nuclear factor-kB (NF-kB), which enhances the expression of adhesion molecules (VCAM-1, ICAM-1) and secretion of proinflammatory cytokines that increase the production of ROS [8]. However, some studies show that, unlike superoxide, other forms of ROS such as hydrogen peroxide (H2O2) produced by NADPH synthase type 4 in endothelial cells, can, on the contrary, prevent the development of atherosclerosis by reducing the secretion of proinflammatory cytokines [9], which expands our understanding of the influence of ROS on the pathogenesis of atherosclerosis. ROS damage to mtDNA leads to the accumulation of mtDNA mutations; a number of studies have shown an association of specific variants of mtDNA heteroplasmy with the manifestation of atherosclerosis [10]. Circulating mtDNA activates the NLRP3 and cGAS–STING inflammatory pathways, promoting the secretion of IL-1β and IL-18 cytokines, enhancing inflammation and atherogenesis [11].

**Figure 1:**
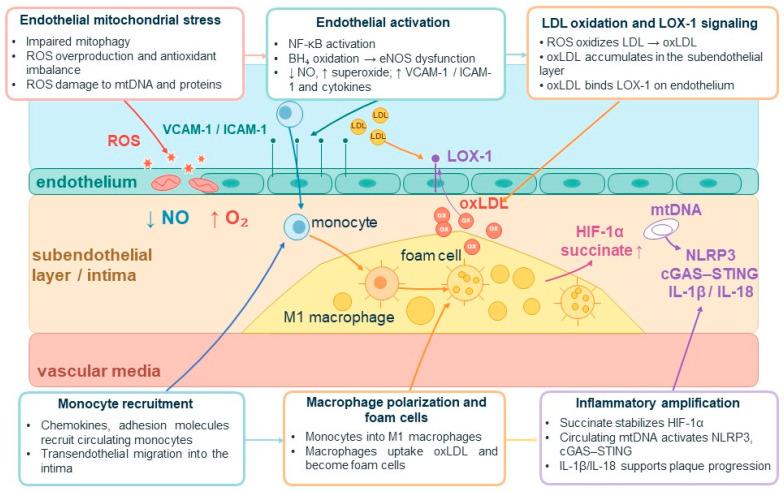
Mitochondrial dysfunction-driven atherosclerotic plaque development. cGAS, cyclic GMP-AMP synthase; eNOS, endothelial nitric oxide synthase; FUS, fused in sarcoma; HIF-1α, hypoxia-inducible factor 1-alpha; ICAM-1, intercellular adhesion molecule 1; IL-1β, interleukin-1 beta; IL-18, interleukin-18; LDL, low-density lipoprotein; LOX-1, lectin-like oxidized low-density lipoprotein receptor-1; M1, classically activated pro-inflammatory macrophage phenotype; mtDNA, mitochondrial DNA; NF-κB, nuclear factor kappa B; NLRP3, NOD-, LRR- and pyrin domain-containing protein 3; oxLDL, oxidized low-density lipoprotein; OXPHOS, oxidative phosphorylation; ROS, reactive oxygen species; RNS, reactive nitrogen species; SOD1, superoxide dismutase 1; STING, stimulator of interferon genes; TARDBP, TAR DNA-binding protein 43 gene; VCAM-1, vascular cell adhesion molecule 1; BH_4_, tetrahydrobiopterin.

In addition to mtDNA oxidation, ROS modify low-density lipoproteins (LDL), and oxidized LDL accumulate in the subendothelial space, binding to the LOX-1 receptor. LOX-1 activation leads to the suppression of eNOS, which impairs NO production and promotes atherosclerosis [12]. Chemokines and adhesion molecules released by endothelial cells during local inflammation attract circulating monocytes from the peripheral blood. In the arterial wall, monocytes differentiate into proinflammatory macrophages (M1), which are characterized by glycolysis as the main mechanism of energy metabolism [13]. In turn, the intensity of oxidative phosphorylation decreases, which leads to the accumulation of succinate, a metabolite of the tricarboxylic acid cycle. The oxidation of succinate causes the increased production of ROS, which activates the transcription factor HIF-1α, increasing the expression of IL-1β [14]. IL-1β promotes the recruitment of new immune cells. Phagocytosis of oxLDL transforms macrophages into foam cells, the basis of atherosclerotic plaques [15]. Figure 1 demonstrates the pathogenetic mechanisms of mitochondrial dysfunction in atherogenesis.

#### 2.1.2. Cardiomyopathy

Cardiomyopathies are a heterogeneous group of diseases associated with abnormal structure and function of the myocardium. The most common type of cardiomyopathy is hypertrophic cardiomyopathy (HCM), a myocardial disease characterized by thickening (hypertrophy) of the left or, less commonly, right ventricle, leading to diastolic dysfunction with preservation of the ejection fraction or its reduction in the terminal stage. HCM is traditionally considered a consequence of mutations in genes encoding sarcomere proteins, such as β-myosin heavy chain (*MYH7*) and myosin-binding protein C (*MYBPC3*). Modern data demonstrate that mitochondrial dysfunction is directly involved in the pathogenesis of HCM [16]. In HCM, decreased activity of electron transport chain proteins is observed in cardiomyocytes, which leads to impaired oxidation in the mitochondria and cellular energy deficiency. In response to ATP deficiency, metabolic reprogramming of cardiomyocytes occurs: the main energy source changes to glycolysis instead of oxidative phosphorylation, which is accompanied by increased expression of the insulin-independent glucose transporter (GLUT1) and glycolytic enzymes [17]. Accumulation of lactate during glycolysis leads to intracellular acidosis, which leads to inhibition of sarcoplasmic reticulum calcium-ATPase (SERCA2a), disrupting calcium metabolism and contributing to myocardial dysfunction [18]. Furthermore, decreased activity of tricarboxylic acid cycle enzymes is observed, which exacerbates the energy crisis. In patients with right ventricular failure, metabolic profiling revealed an accumulation of intermediate metabolites of the tricarboxylic acid cycle, indicating impaired oxidation processes in the myocardium [19].

As described previously, dysfunction of the electron transport chain leads to increased production of ROS, which damage mtDNA and mitochondrial membranes. Dysregulation of mitophagy pathways, particularly the PINK1/Parkin ubiquitin-dependent pathway, leads to the accumulation of damaged mitochondria that produce ROS [20]. In addition to dysregulation of mitophagy pathways, mitochondrial dynamics is disrupted, and the expression of mitofusins (MFN1, MFN2) and the OPA1 protein, responsible for internal membrane fusion and cristae organization, is reduced. In turn, disrupted mitochondrial dynamics leads to organelle fragmentation [21]. Mitochondrial dysfunction can result from mutations in genes encoding mitochondrial proteins, both in the nuclear genome (*ELAC2*, *NDUFAF1*) [22] and in the mitochondrial genome (*MT-TL1*, *MT-CYB*) [23]. Electron transport chain proteins are encoded by both the nuclear and mitochondrial genomes, requiring more precise regulation of expression for normal subunit assembly and ATP production. Certain mitochondrial haplogroups have been shown to alter the clinical manifestations of pathogenic nuclear DNA mutations [24].

Mitochondrial dysfunction contributes to the opening of the mitochondrial permeability transition pore (MPTP), loss of membrane potential, and the release of cytochrome c into the cytoplasm, triggering apoptotic cascades, which contributes to myocardial fibrosis [25]. Mutations associated with the structure and function of sarcomeres indirectly affect the normal functioning of mitochondria. Mutations in the contractile apparatus increase ATP consumption, which contributes to mitochondrial overload and the progression of oxidative stress [26].

### 2.2. Metabolic Syndrome

Metabolic syndrome is a complex of interrelated metabolic disorders that significantly increases the risk of cardiovascular disease, type 2 diabetes, and obesity. At the cellular level, metabolic syndrome is a disruption of energy homeostasis. Mitochondrial dysfunction leads to oxidative stress and impaired energy production, which is the primary cause of metabolic syndrome [27].

#### 2.2.1. Type 2 Diabetes

Type 2 diabetes mellitus is a chronic disease characterized by insulin resistance, leading to elevated blood glucose levels. Mitochondrial dysfunction is one of the key mechanisms contributing to the development of insulin resistance and impaired insulin secretion in pancreatic β-cells. It acts through insulin resistance through several mechanisms: oxidative stress, accumulation of lipid metabolism intermediates, and decreased ATP production via oxidative phosphorylation, which critically affects insulin signaling. Studies have shown that elevated insulin levels increase ATP production by 30% in healthy individuals, whereas in patients with type 2 diabetes, no changes in ATP synthesis are observed despite comparable insulin levels [28]. This is due to the fact that the cell lacks energy to activate key stages of insulin signaling, including insulin receptor substrate 1 (IRS-1), the main mediator in signal transmission from the insulin receptor into the cell, and AKT, a kinase that regulates the translocation of the GLUT4 glucose transporter to the cell membrane, which ensures glucose uptake by the cell. It was shown that the reduction in oxidative stress leads to restored GLUT4 translocation [29].

Increased levels of ROS disrupt insulin signaling components through several mechanisms: first, oxidative stress disrupts insulin signaling primarily through redox modifications of cysteine residues in downstream components of the cascade (protein tyrosine phosphatases, kinases), which leads to decreased phosphorylation of the insulin receptor [30]. Second, oxidative stress activates the stress kinase JNK, which phosphorylates IRS-1 at serine residues, inhibiting signal transduction [31]. Third, oxidation of cell membrane lipids and cholesterol under the influence of ROS disrupts the localization of receptors in lipid rafts [32], and consequently, the insulin receptor. In addition, ROS damage mtDNA and mitochondrial membranes, leading to the release of oxidized mtDNA into the cytoplasm, where they act as damage-associated molecular patterns (DAMPs), activating the NLRP3 inflammasome [10] and the NF-κB inflammatory pathway, promoting the production of inflammatory cytokines, including IL-1β, IL-6, and TNF-α, which activate serine kinases that phosphorylate IRS-1 at inhibitory sites, contributing to insulin resistance [33].

Mitochondrial dysfunction also disrupts β-oxidation of fatty acids, leading to the accumulation of lipids and lipid metabolism intermediates. It was shown that in white adipose tissue mitochondrial dysfunction leads to the accumulation of sphingolipids, primarily ceramides, which activate protein phosphatases that dephosphorylate key components of the insulin cascade, including AKT [34].

In addition to insulin resistance, type 2 diabetes mellitus also exhibits reduced secretory activity of pancreatic β-cells, which is also associated with mitochondrial dysfunction [35]. This is due to several factors, such as insufficient ATP production, oxidative stress, and impaired calcium homeostasis. Cellular stress disrupts oxidative phosphorylation and, consequently, ATP production. This disrupts the function of ATP-sensitive potassium channels (KATP channels), which play a key role in insulin secretion by regulating transmembrane potential [36]. Reduced ATP production and oxygen consumption can be caused by the mtDNA A3243G mutation, as it disrupts mitochondrial translation and assembly of electron transport chain complexes, contributing to pancreatic β-cell dysfunction [37].

Disruption of calcium homeostasis is a consequence of cellular stress caused by mitochondrial dysfunction due to ROS and mitochondrial DAMPs. The endoplasmic reticulum is the main calcium depot in the cell. Oxidative stress causes oxidation of endoplasmic reticulum channel proteins by reactive oxygen species, resulting in calcium leakage into the cytoplasm [38]. Calcium is actively transported into the mitochondria via the VDAC and MCU channels [39], causing disruption of the transmembrane potential and normal mitochondrial functioning, and, as a consequence, the synthesis of excess ROS, the opening of the MPTP and the release of cytochrome c into the cytoplasm, the initiation of apoptosis [40], and the death of pancreatic β-cells.

#### 2.2.2. Obesity

Obesity is a fundamental component of metabolic syndrome associated with cardiovascular disease and type 2 diabetes. Mitochondrial dysfunction underlies this association, linking obesity, insulin resistance, and chronic inflammation [27].

Adipose tissue is an important endocrine organ regulating metabolism throughout the body, but its function is impaired in obesity. Several studies demonstrate a difference between the mtDNA content in visceral and subcutaneous adipose tissue: the mtDNA content is 2-fold lower in visceral adipose tissue, indicating reduced mitochondrial activity. In insulin-resistant individuals, the mtDNA copy number in visceral adipose tissue is significantly reduced compared to control (*p* = 0.0317), and this reduction is statistically significantly correlated with body mass index (R^2^ = −0.57) and the QUICKI insulin sensitivity index (R^2^ = 0.51), suggesting that decreased mitochondrial activity precedes or accompanies insulin resistance [28].

Excessive nutrient intake overloads the Krebs cycle and causes oxidative stress and inflammation, damaging mitochondrial proteins and DNA and leading to organelle dysfunction. In adipose tissue, this is manifested by a decrease in the expression of key genes of the insulin cascade encoding IRS-1 and AKT, as well as genes encoding lipid metabolism proteins such as PPARG, SIRT1 and LPL, which directly correlates with a decrease in mtDNA copies [28]. In addition, there is a positive correlation between the number of mtDNA copies and the expression of epigenetic regulators (HDAC2, DNMT1, DNMT3A) that affect the functioning of mitochondria, indicating metabolic reprogramming. Thus, mitochondrial dysfunction in adipose tissue is not limited to local effects, but triggers a cascade of systemic disorders such as insulin resistance, inflammation and progression of associated diseases.

#### 2.2.3. Non-Alcoholic Fatty Liver Disease

Non-alcoholic fatty liver disease (NAFLD) is a common manifestation of metabolic syndrome. Mitochondrial dysfunction of hepatocytes is considered a central mechanism of NAFLD, which implies the transition of the simple steatosis to steatohepatitis and further to fibrosis and cirrhosis. In NAFLD, hepatic metabolic function is altered under the influence of obesity and excessive carbohydrate load, which leads to increased *de novo* lipogenesis in the liver. According to studies, the contribution of *de novo* lipogenesis to palmitate synthesis leads to a 3-fold increase in NAFLD [41]. Together with the increased influx of non-esterified free fatty acids into the liver, obtained during lipolysis of adipose tissue, both of these factors contribute to fatty acid overload in the liver [42].

Mitochondria adapt to the stress by increasing the import and oxidation of free fatty acids, enhancing β-oxidation. This leads to metabolic stress and increased ROS production, which activate NF-κB and the NLRP3 inflammasome [10,11], triggering the production of proinflammatory cytokines that attract monocytes from the peripheral blood into the liver parenchyma, causing steatohepatitis. It is worth noting that in fatty liver disease, the intensity of mitochondrial processes increases; however, the efficiency of oxygen oxidation is reduced, leading to the progression of oxidative stress and inflammation, as ROS induce mitochondrial DAMPs. Normally, when fatty acids are in excess, the liver converts acetyl-CoA into ketone bodies (β-hydroxybutyrate), which are used as an energy source by other tissues, thereby relieving the lipid overload. In NAFLD this mechanism is disrupted since β-hydroxybutyrate levels negatively correlate with liver fat content [43]. Acetyl-CoA is not excreted from the liver but is directed into the tricarboxylic acid cycle and *de novo* lipogenesis, leading to increased ROS production, inflammation and the development of steatohepatitis and fibrosis [44].

### 2.3. Neurodegenerative Diseases

**Figure 2:**
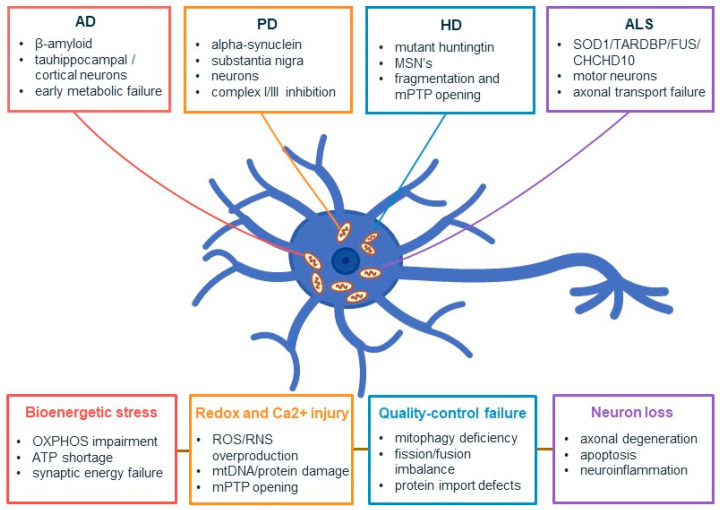
Mitochondrial dysfunction in the pathogenesis of neurodegeneration. AD, Alzheimer’s disease; PD, Parkinson’s disease; HD, Huntington’s disease; ALS, amyotrophic lateral sclerosis; MSN’s, Medium Spiny Neurons; ATP, adenosine triphosphate; CHCHD10, coiled-coil-helix-coiled-coil-helix domain-containing protein 10; mPTP, mitochondrial permeability transition pore.

Mitochondrial dysfunction is involved in pathogenetic mechanism of neurodegenerative diseases, including Alzheimer’s disease, Parkinson’s disease, amyotrophic lateral sclerosis, and Huntington’s disease [45]. The most common clinical manifestations of primary mitochondrial disorders are neurological and neuromuscular syndromes. The central nervous system is characterized by an exceptional metabolic load: with a mass of approximately 2% of body weight, the brain consumes approximately 25% of glucose and 20% of all oxygen entering the body, both during wakefulness and at rest [46]. The lifespan of a neuron is equivalent to the lifespan of the entire organism and requires a constantly high level of ATP synthesis to maintain membrane potential, synaptic transmission, and axonal transport. Neurons of the cerebral cortex at rest consume 4.7 billion ATP molecules per second, or approximately 6 kg of ATP per day [47]. Accordingly, neurons contain a significant mass of mitochondria, localized in the cell body, axon, and especially in the presynaptic terminals. Mitochondrial dynamics (transport, fusion, fission, mitophagy) are essential for maintaining energy homeostasis in nervous tissue [48]. In addition to ATP production through oxidative phosphorylation, mitochondria are involved in the regulation of calcium homeostasis, the biosynthesis of iron–sulfur clusters and heme, the synthesis of neurotransmitters, the control of apoptosis, and the generation of reactive oxygen species (ROS) [48]. The high intensity of oxidative metabolism combined with relatively limited antioxidant defense makes neurons particularly vulnerable to oxidative stress and bioenergetic failure [49]. It has been established that disturbances in mitochondrial dynamics, respiratory chain defects, a decrease in the number of mtDNA copies, suppression of biogenesis, impaired import of nuclear-encoded proteins, and mitophagy deficiency are associated with the progression of neurodegenerative processes [50]. It is assumed that mitochondrial dysfunction increases the vulnerability of neurons to disease-specific factors and largely determines the topography of damage in various nosologies [51]. Figure 2 demonstrates the key mechanisms of mitochondrial dysfunction in the pathogenesis of neurodegenerative diseases.

Alzheimer’s disease (AD) is the most common cause of dementia, accounting for 60–70% of cases. Morphologically, the disease is characterized by extracellular accumulation of β-amyloid and intracellular formation of neurofibrillary tangles from hyperphosphorylated tau protein [52]. Impaired energy metabolism is recognized as one of the earliest and most persistent signs of AD. In the brains of patients, decreased activity of respiratory chain complexes, a decrease in the number of mtDNA copies, suppression of mitochondrial biogenesis signaling pathways (in particular, in pyramidal neurons of the hippocampus), and pronounced morphological abnormalities of mitochondria are detected [53]. Oxidative stress, characteristic of vulnerable neuronal populations, additionally disrupts the import of nuclear-encoded mitochondrial proteins, aggravating the energy deficit [54]. Two complementary concepts are discussed in the pathogenesis of AD. According to the amyloid cascade hypothesis, the accumulation of β-amyloid and tau induces secondary mitochondrial dysfunction. Within the framework of the mitochondrial cascade hypothesis, the primary event is a disruption of mitochondrial metabolism, and amyloidogenesis is considered a consequence of energy and oxidative imbalance [55]. Regardless of the sequence of events, mitochondrial dysfunction is recognized as a key link in the pathogenesis of both familial and sporadic forms of the disease [56]. Thus, in Alzheimer’s disease, mitochondria act not only as a target of pathological processes, but also as an active modifier of neurodegeneration, determining early bioenergetic deficiency, oxidative damage, and progressive neuronal death.

Parkinson’s disease (PD) is a progressive neurodegenerative disorder with a prevalence of up to 1% in individuals under 45 years of age and up to 2% in older individuals [57]. Morphologically, PD is characterized by selective loss of dopaminergic neurons in the substantia nigra pars compacta and the formation of intracellular aggregates of α-synuclein (Lewy bodies) [58]. Mitochondrial dysfunction is considered to be one of the central links in the pathogenesis of PD [59]. The most reproducible biochemical disorder is the inhibition of complexes I and III of the respiratory chain, accompanied by decreased ATP synthesis and excessive production of reactive oxygen species [59]. Oxidative stress, in turn, promotes pathological aggregation of α-synuclein [60]. The interaction of α-synuclein and mitochondria is two-way. Excessive or mutant α-synuclein is able to bind to proteins of the mitochondrial protein import complex, as well as to components of the respiratory chain and ATP synthase, causing depolarization of the inner membrane and a further decrease in the activity of complex I. Simultaneously, mitochondrial dysfunction disrupts the degradation of amyloidogenic proteins and increases their cytosolic accumulation. Thus, a self-sustaining pathological cycle of “oxidative stress—α-synuclein aggregation—respiratory chain inhibition” is formed. Impairment of mitophagy and the proteostasis system is of additional significance [61]. Defects in autophagy and the lysosomal pathway, including dysregulation of the PINK1/Parkin signaling cascade, impede the removal of damaged mitochondria, which increases energy deficiency and oxidative damage [62]. The selective vulnerability of dopaminergic neurons is explained by their high metabolic load, intense calcium dynamics, and the additional formation of reactive oxygen species during dopamine metabolism [63]. Taken together, this makes these neuronal populations particularly sensitive to disturbances in mitochondrial homeostasis. Regardless of whether mitochondrial dysfunction is a primary or secondary event, it is a key factor in the progression of PD.

Huntington’s disease is an autosomal dominant progressive neurodegenerative disorder caused by the expansion of CAG repeats in the HTT gene. The expansion of the repeats results in the synthesis of mutant huntingtin (mHTT) with an extended polyglutamine region; the length of the CAG repeat is inversely correlated with the age of disease onset [64]. The most pronounced neurodegeneration in Huntington’s disease is localized in the striatum (caudate nucleus and putamen) and the cerebral cortex. GABAergic medium spiny neurons are primarily affected, with neurons of the indirect pathway (D2R+) degenerating earlier than neurons of the direct pathway, which determines the characteristic sequence of motor manifestations of the disease [65]. Mitochondrial dysfunction in HD is caused by direct interaction of mutant huntingtin with mitochondrial outer membrane proteins (TOM20, Drp1, VDAC), which causes fragmentation of the mitochondrial network, decreased membrane potential, defective import of nuclear-encoded proteins, and increased sensitivity to Ca^2+^-induced opening of mPTP with the release of cytochrome c. The combination of these disturbances leads to energy deficiency and activation of apoptotic mechanisms [64,65]. Mitochondrial dysfunction is also accompanied by excessive production of ROS/RNS, the formation of oxidative and nitrosative stress with damage to mtDNA (increased frequency of deletions). Calcium imbalance (via NMDA receptors), activation of caspases, and defects in axonal transport aggravate the pathology, leading to mitochondrial failure [66]. There is evidence that mitochondrial dysfunction is detected already at the preclinical stage in mutation carriers, which allows us to consider it as an early component of pathogenesis [64]. Overall, the combination of experimental and clinical data indicates that mitochondrial dysfunction is a system-forming factor in neurodegeneration in Huntington’s disease, determining energy deficiency, oxidative damage, and neuronal death.

Amyotrophic lateral sclerosis (ALS) is a rapidly progressive, fatal neurodegenerative disease characterized by the death of upper and lower motor neurons with the development of progressive paralysis [67]. In recent years, mitochondrial dysfunction has been considered a systemically important link in the pathogenesis of ALS. Clinically, ALS is associated with pronounced metabolic disturbances: hypermetabolism, weight loss, and changes in the lipid profile [68]. These phenomena reflect a systemic energy imbalance and indirectly indicate mitochondrial insufficiency. At the morphological level, dense accumulations of mitochondria in the anterior horns of the spinal cord, presynaptic swelling of organelles in motor neurons, and their abnormal distribution—predominant localization in the soma and proximal axon with a deficit in the distal regions—have been described [69]. A decrease in the expression of the Miro1 protein, which regulates mitochondrial transport along microtubules, further confirms the impairment of axonal delivery of organelles [70]. A number of genes associated with ALS (*SOD1*, *TARDBP*, *FUS*) are directly or indirectly involved in the regulation of mitochondrial homeostasis [71,72]. It has been shown that mutant SOD1 is localized in the intermembrane space of mitochondria, disrupting the functioning of the respiratory chain and increasing the production of reactive oxygen species [72]. Of particular importance is the identification of mutations in the *CHCHD10* gene associated with mitochondrial DNA breakpoint syndrome. The *CHCHD10* protein is localized in the intermembrane space of mitochondria and belongs to the coil-coil-helix protein family. It is assumed that it is involved in maintaining the structure of the cristae and regulating mitochondrial fusion through interaction with OPA1. Mutations in *CHCHD10* result in disruption of the inner membrane architecture, respiratory chain defects, and increased sensitivity to apoptotic stimuli [73]. Motor neurons have a very high energy requirement, making them particularly dependent on efficient axonal transport of mitochondria and local ATP synthesis. Disruption of these processes leads to energy deficiency at neuromuscular synapses, axonal degeneration, and subsequent neuronal death. Dysfunction of the respiratory chain is accompanied by excessive production of reactive oxygen species, damage to mtDNA, and inner membrane proteins. Increased mitochondrial membrane permeability and cytochrome c release activate caspase-dependent apoptotic pathways. A parallel neuroinflammatory response develops, which exacerbates mitochondrial damage and creates a vicious cycle of neurodegeneration. A combination of clinical, morphological, and molecular data suggests that mitochondrial dysfunction is an early and pathogenetically significant component of ALS. Impaired energy metabolism, mitochondrial dynamics and transport, defects in cristae and mitophagy, and oxidative stress form a cascade of events leading to selective motor neuron death. Therefore, mitochondria are considered not only a target for secondary damage but also a potential therapeutic target in ALS.

### 2.4. Oncology

Mitochondria in malignant cells are currently viewed not as functionally suppressed organelles subject to glycolysis, but as highly dynamic metabolic hubs that support tumor initiation, progression, immune suppression, and resistance to therapy. Recent metabolomic, fluxomic, and single-cell studies have shown that mitochondrial dysfunction in cancer is not synonymous with bioenergetic failure. Instead, it reflects context-dependent alterations in oxidative phosphorylation (OXPHOS), tricarboxylic acid cycle (TCA) activity, redox signaling, and biosynthetic fluxes [74].

Although the classical Warburg effect suggests a shift towards aerobic glycolysis, numerous studies published in recent years indicate that many solid tumors and hematological malignancies retain the capacity for oxidative phosphorylation and, in some cases, even enhance it [75]. For example, the cancer suppressor gene p53 suppresses glycolysis and promotes oxidative phosphorylation in mitochondria through a number of downstream targets, antagonizing the Warburg effect [76]. Furthermore, recent functional screens using CRISPR technology targeting nuclear-encoded mitochondrial genes have identified components of complex I of the respiratory chain, the mitochondrial translation system, and one-carbon metabolism as factors required for proliferation under certain oncogenic conditions [77]. These data suggest that mitochondrial metabolism is selectively regulated depending on oncogenic factors and tissue location, supporting the concept of metabolic context dependence rather than uniform mitochondrial suppression. Another manifestation of energy plasticity is associated with epigenetic regulation under the influence of metabolites. Accumulation of intermediates of the tricarboxylic acid cycle, such as succinate, fumarate, and 2-hydroxyglutarate (2-HG), often caused by mutations in the genes of succinate dehydrogenase (SDH), fumarate dehydrogenase (FH), or isocitrate dehydrogenase 1/2 (IDH1/2), leads to inhibition of α-ketoglutarate-dependent dioxygenases [78]. It has been shown that in gliomas with mutations in the IDH genes, there is a restructuring of mitochondrial metabolism, which directly affects chromatin structure and the state of differentiation, linking mitochondrial dysfunction with oncogenic epigenetic reprogramming [79].

Hypoxia is a hallmark of the tumor microenvironment and a powerful factor influencing mitochondrial remodeling. Stabilization of HIF-1α and HIF-2α proteins reprograms mitochondrial metabolism by suppressing pyruvate dehydrogenase by increasing pyruvate decarboxylase1 expression, regulating complex IV composition, and promoting mitophagy. However, recent data indicate that hypoxia not only reduces mitochondrial activity but also significantly alters it [80]. Proteomic and respiratory analysis of hypoxic tumor spheroids revealed selective remodeling of respiratory chain supercomplexes, optimizing electron transfer efficiency and limiting excessive reactive oxygen species formation. Hypoxia-induced *COX7A2L* expression was shown to promote adaptive turnover of complexes I and IV, preserving bioenergetic potential at low oxygen partial pressure [81]. Mitochondrial ROS serve signaling functions in addition to being toxic byproducts. Recent studies using genetically encoded redox sensors in vivo have shown that acute ROS exposure stabilizes the HIF signaling pathway and activates pro-oncogenic pathways such as NF-κB and MAPK in hypoxic tumor sites. This redox-dependent signaling pathway supports angiogenesis, epithelial–mesenchymal transition, and tumor metastasis [82]. Hypoxia also induces selective mitophagy through the activation of BNIP3 and NIX proteins. Functional studies in pancreatic cancer models have shown that hypoxia-induced mitophagy impairment leads to the accumulation of dysfunctional mitochondria, increased oxidative stress, and decreased tumor growth in vivo [83]. This suggests that mitochondrial quality control is a critical adaptive mechanism rather than a passive response.

Remarkably, metabolic compartmentalization occurs within tumors: hypoxic cores exhibit elevated levels of glycolytic processes and signs of mitophagy, while invasive fronts exhibit elevated levels of oxidative phosphorylation and markers of mitochondrial biogenesis. This spatial heterogeneity suggests the coexistence of different mitochondrial states, which lead to distinct metabolic phenotypes, within a single tumor [84].

Thus, the existing understanding of the role of mitochondrial dysfunction in the development of socially significant chronic diseases was summarized. At the same time, these diseases are multifactorial and may be associated with other equally important factors, such as inflammation, genetics, metabolism, aging, environmental factors, and tissue-specific mechanisms. However, despite recent advances in the development of diagnostic and therapeutic approaches for a number of these pathologies, the studies of the pathogenetic mechanisms and search for new treatment targets remain extremely relevant. Therefore, the study of mitochondrial dysfunction biomarkers and their integration into clinical practice is of particular interest for modern research.

## 3. Modern Approaches to Study Mitochondrial Dysfunction

**Figure 3:**
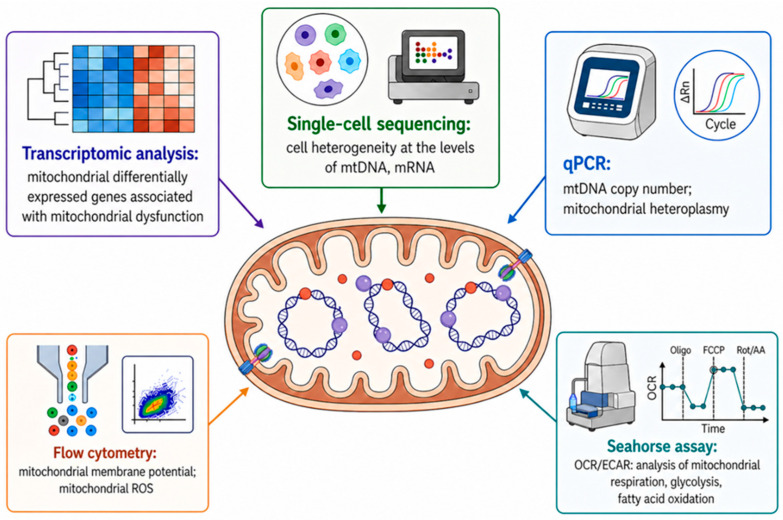
Modern methods for diagnosing mitochondrial dysfunction.

The importance of mitochondrial dysfunction in the pathogenesis of various diseases makes the development of diagnostic tests aimed to investigate mitochondrial function a highly relevant area of modern science. Modern approaches to diagnosing mitochondrial dysfunction include genetic studies and instrumental methods that allow assessing cellular energy metabolism (Figure 3).

### 3.1. Transcriptome Analysis

Transcriptome analysis is one of the key methods in studying mitochondrial dysfunction, as it allows us to evaluate the changes in gene expression associated with mitochondrial metabolism and the cellular stress response [85,86,87]. The results of transcriptome analysis have identified a number of mitochondrial differentially expressed genes (mitoDEGs) that can be considered as biomarkers for assessing mitochondrial dysfunction before clinical manifestation. Table 1 presents the key mitoDEGs that may serve as biomarkers of mitochondrial dysfunction.

*MTHFD2* is one of the most studied differentially expressed genes associated with mitochondrial dysfunction. It is involved in maintaining mitochondrial redox homeostasis through NADPH metabolism [88]. *MTHFD2* codes mitochondrial enzyme methylenetetrahydrofolate dehydrogenase 2 (MTHFD2), essential for one-carbon metabolism. Numerous studies have shown altered *MTHFD2* expression in various diseases. The upregulation of *MTHFD2* was demonstrated in a murine model of intracerebral hemorrhage. In this study it has been shown that mice with knockdown of *MTHFD2* are characterized by increased apoptosis and worsened cognitive impairment after intracerebral hemorrhage [88]. In glioblastoma, *MTHFD2* expression was significantly increased in glioblastoma tissues compared with adjacent normal tissues; macrophages with high levels of *MTHFD2* expression demonstrated significant activation of signaling pathways associated with inflammation, hypoxia, glycolysis, and tumor progression, in particular NF-κB and IL6/JAK/STAT3 [89]. The expression of *Mthfd2* along with *Vwa8* and *Decr1* genes was also significantly increased in samples of left ventricle tissue in mice with heart failure with preserved ejection fraction [90]. The folate metabolism regulated by MTHFD2 is involved in the development and progression of several malignancies. It was demonstrated that *MTHFD2* expression was increased in samples of kidney cancer and lymph node metastasis so it may serve as a prognostic marker for kidney renal clear cell carcinoma along with serum folate level [91]. MTHFD2 is also required for TGF-β-induced glycine synthesis in human lung fibroblasts that is an important pathogenetic mechanism for collagen production in the development of idiopathic pulmonary fibrosis [92]. Elevated MTHFD2 was observed in patients with rheumatoid arthritis and in a murine model of arthritis and correlated positively with osteoclastogenesis [103]. At the same time, inhibition of MTHFD2 suppressed fibrotic responses of cultured human lung fibroblasts and decreased osteoclast formation and ameliorated bone loss in mice with collagen-induced arthritis [92,104].

*BCL2* codes mitochondrial outer membrane protein B-cell lymphoma 2 (BCL2) that suppresses apoptosis. BCL2 protein controls the permeability of the outer mitochondrial membrane and the release of cytochrome c and other apoptotic factors. Dysregulation of the mitochondrial apoptotic pathway reflects mitochondrial stress and indicates a manifestation of mitochondrial dysfunction [103]. Transcriptome analysis [87] identified *BCL2* and a number of other genes, including *FOXO1* as potential biomarkers for chronic obstructive pulmonary disease [87]. Post-transcriptional regulation of *BCL2* expression via microRNA, in particular, microRNA-1, plays an important role in cardiomyocyte apoptosis after myocardial ischemia-reperfusion injury and in the pathogenesis of neurodegenerative diseases, so it can be considered as a potential treatment approach to develop a pharmacological intervention aimed at the modulation of apoptosis pathways [93,94]. Transcription factor forkhead box protein O1 (FOXO1) is one of the key metabolic regulators in the cell, involved in the cellular response to oxidative stress, in the regulation of the cell cycle and apoptosis, and glucose and lipid metabolism. Regarding mitochondrial quality, FOXO1 controls mitochondrial dynamics (mitochondrial fission and fusion) and the expression of genes responsible for mitophagy [95]. A number of studies have shown the important role of FOXO1 in the metabolism and mitochondria functions of cardiomyocytes in cardiovascular and metabolic diseases [95]. The development of therapeutic approaches based on microRNAs regulating FOXO1 is also a promising area of research in cardiology [95].

Since mitochondria play a central role in cellular energy metabolism, altered expression of genes associated with mitochondrial oxidative phosphorylation (OXPHOS) is important for studying mitochondrial dysfunction. Previous studies have shown that mitochondrial dysfunction is accompanied by activation of OXPHOS gene expression to maintain energy metabolism; however, long-term mitochondrial dysfunction leads to decreased OXPHOS gene expression, indicating decompensation [105]. In particular, mitochondrial dysfunction is associated with increased expression of genes encoding subunits of the mitochondrial respiratory chain complex such as ubiquinol-cytochrome c reductase (*UQCR*), NADH: ubiquinone oxidoreductase flavoprotein (*NDUF*), cytochrome c oxidase (*COX*), and ATP synthetase complex V (*ATP5*) [96,97,106]. In vivo studies and study in patients with myocardial ischemia-reperfusion injury have shown that activation of the *S100A9* gene, one of the key factors in myocardial damage, is associated with downregulation of *NDUF*, *COX* and *ATP5*, followed by the development of pathological changes in the myocardium due to mitochondrial dysfunction [96,97].

Mitochondrial transcription factor A (TFAM) regulates mtDNA transcription, replication, and reparation [107]. In addition, TFAM is involved in the packaging of mtDNA, organizing it into nucleoids, and thus controlling the expression of mtDNA genes [108]. Reduced *TFAM* expression is primarily associated with decreased mtDNA copy number disruption of the mitochondrial respiratory chain due to enzyme deficiency caused by the restriction of mtDNA transcription. A number of studies demonstrate a relationship between changes in *TFAM* expression and mitochondrial dysfunction. Thus, it was shown that polystyrene microplastics induce the development of mitochondrial dysfunction in cultured C2C12 myotubes, namely, it reduces the expression of *TFAM* and OXPHOX genes, causes changes in mitochondrial morphology—swelling and destruction of cristae, a decrease in mitochondrial membrane potential and ATP production [98]. A study in broilers with pulmonary hypertension syndrome demonstrated that hypoxia and oxidative stress stimulate mitochondrial biogenesis through the activation of the PGC-1α/Nrf-s/Tfam pathway, which results in an increase in mitochondrial number and more ATP production [99]. TFAM is used as a marker to assess the effects of various preparations and biologically active substances on mitochondrial function. For example, it has been shown that telmisartan, epicatechin, terpinen-4-ol, and oxytocin ameliorate mitochondrial dysfunction in various experimental models via upregulation of *TFAM* expression [99,109,110,111]. Animal models with *TFAM* knockdown/knockout are used in experimental studies of mitochondrial dysfunction [111,112]. It was shown in *TFAM* knockout mice that systemic TFAM deficiency leads to a reduction in the number of mitochondria in different tissues, which results in subsequent multiorgan dysfunction and premature death [112]. The therapeutic potential of small-molecule TFAM activators is currently being studied for diseases associated with mitochondrial dysfunction. These compounds promote an increase in TFAM secretion and mtDNA copy number [113].

MitoDEGs also include genes encoding carrier proteins that mediate the transport of metabolites across the mitochondrial membrane. Mitochondrial transporters solute carrier family 25 members (SLC25A) are responsible for the transfer of ATP, amino acids, organic acids and other cofactors such as NAD+, coenzyme A derivatives, etc., between the mitochondrial and cytosolic compartments [114]. It has been shown that *SLC25* family gene expression was elevated in the brains of mice with depression-like disorder and correlated with mitochondrial *Mrps* and *Mrpl* gene expression, which increases due to the load on the mitochondrial respiratory chain [115].

Finally, mitochondrial health is reflected by mitoDEGs associated with mitochondrial dynamics. In particular, it was shown in a mouse model with hepatic steatosis that mitochondrial dysfunction increases the expression of the *OPA1* gene, encoding mitochondrial dynamin like GTPase, a protein of the inner mitochondrial membrane involved in mitochondrial fusion, which leads to a decrease in oxidative stress [101]. Another study aiming to analyze the transcriptome data of patients with atherosclerosis and type 2 diabetes revealed the strong association in the expression of *mitoDEGs MTF1*, encoding mitofusin responsible for mitochondrial fusion, and *DRP1*, encoding dynamin-related protein 1 responsible for mitochondrial fission with key pathogenetic factors of these diseases including mitochondrial metabolism [115]. The balance of mitochondrial fusion and fission processes is important in the pathogenesis of a wide variety of diseases associated with chronic inflammation, aging, autoimmunity, and genetic disorders. The analysis of mitochondrial dysfunction by transcriptome analysis also includes the evaluation the *PINK1* and *Parkin* genes, markers of mitophagy activation. Mitophagy is a protective response of the cell to mitochondrial damage, which is currently considered as a potential therapeutic target in diseases associated with mitochondrial dysfunction. A transcriptomic analysis of a model of mice with hepatic steatosis revealed that treatment aimed at amelioration of fatty acid β-oxidation and mitochondrial function is associated with activation of PINK1/Parkin-dependent mitophagy [102]. The study of PINK1-Parkin-dependent mitophagy is of particular importance for the study of pathogenesis and the development of diagnostic and therapeutic strategies for neurodegenerative diseases, namely Parkinson’s disease [116]. It is known that PINK1/Parkin-dependent mitophagy is activated in the early stages of Parkinson’s disease, and disease progression is characterized by impaired mitophagy and the accumulation of dysfunctional mitochondria. The transcriptome analysis in a cellular model of Parkinson’s disease demonstrated the upregulation of the *PINK1/Parkin* gene, which decreased with cannabinoid therapy, considered as a new therapeutic strategy against neurodegenerative diseases [117].

Thus, transcriptome analysis allows us to identify a number of mitoDEGs that reflect key signaling pathways in the pathogenesis of various pathological conditions associated with mitochondrial dysfunction, and can be considered as promising targets for the development of novel diagnostic and therapeutic strategies. qPCR analysis and Western blotting protein detection may be used for quantitative verification of the expression of mitoDEGs and development of diagnostic tests. At the same time, transcriptome analysis does not reflect the functional activity of proteins encoded by mitoDEG, and therefore does not allow us to assess the metabolic potential of mitochondria. In this regard, to study mitochondrial dysfunction, it is advisable to use transcriptome data in accordance with other research methods.

### 3.2. Seahorse Assay

The Seahorse assay is an extracellular flux assay that allows investigating the key parameters of mitochondrial function such as glycolysis, mitochondrial respiration, and fatty acid oxidation in real time [118]. The analysis is based on the assessment of oxygen consumption rate (OCR) and extracellular pH that reflects extracellular acidification rate (ECAR). The Seahorse platforms are used for assessment of mitochondrial respiration parameters and stress tests with oligomycin, which blocks ATP synthase in the mitochondrial electron transport chain, FCCP, which uncouples oxidative phosphorylation by dissipating the proton gradient, and rotenone/antimycin A, an inhibitor of the electron transport chain, in isolated mitochondria or cells, revealing mitochondrial dysfunction in various pathologic conditions [119]. The Seahorse analysis of mitochondrial function has demonstrated that parameters of mitochondrial respiration were elevated, while spare capacity and the cell’s ability to response to unexpected energetic stress were decreased in peripheral blood mononuclear cells of patients with Parkinson disease in comparison with heathy subjects [120]. Seahorse assay revealed the association of changes in mitochondrial respiration in platelet and skeletal muscle tissue in a murine model of type 2 diabetes compared to healthy C57BL/6J mice, indicating that platelet bioenergetic profiles mirror the metabolic status of skeletal muscle in healthy and genetically diabetic mice [121]. Intracellular calcium homeostasis is important for normal mitochondrial function because cytosolic calcium regulates mitochondrial ATP production via the tricarboxylic acid cycle and electron transport chain, and thus can be indirectly assessed by the Seahorse method [122]. Despite the high sensitivity and real-time analysis, the Seahorse method has a number of significant limitations when studying mitochondrial dysfunction. First, it is characterized by significant data variability due to technical issues, which requires strict data quality control. Secondly, the analysis of cultured cells does not reflect the specific features of intercellular interactions. Furthermore, the duration of the experiment limits the analysis of long-term effects and requires additional proteomic data and mtDNA measurements for accurate interpretation of the results [118].

### 3.3. qPCR: mtDNA Copy Number and Heteroplasmy

qPCR for mtDNA allows evaluating the copy number of mtDNA and the presence of mtDNA mutations to assess the level of mitochondrial heteroplasmy. Currently, circulating mtDNA copy number is considered as a biomarker of chronic diseases associated with mitochondrial dysfunction. Changes in mtDNA copy number in circulating monocytes have been demonstrated in patients with cardiovascular disease [123,124], obesity [124], rheumatic diseases [125,126], cancer [127], and other pathologies [128]. At the same time, several studies demonstrate no correlation between mtDNA copy number and cancer development [129,130]; controversial data have also been obtained for neurodegenerative diseases [131]. It was shown that leukocyte mtDNA copy number correlated with hs-CRP serum level in older adults, reflecting the important role of mitochondria in the pathogenesis of inflammageing [132]. Studies in various pathologies demonstrate both increases and decreases in mtDNA copy number not only in monocytes, but also in other cell types, since different tissue types have different energy requirements and different mitochondrial content. This is explained by the fact that an increase in mtDNA copy number often reflects a compensatory response to mitochondrial dysfunction, meaning the cell attempts to maintain energy metabolism by increasing the number of mitochondria. A decrease in the copy number of mtDNA usually characterizes later stages of the disease, when severe mitochondrial damage and impaired biogenesis are observed. Transcriptome analysis of HEK 293T cell line showed that a decrease in mtDNA copy number leads to the activation of signaling pathways aimed at restoring mitochondrial function, in particular, mtDNA replication, oxidative phosphorylation and glycolysis [133].

The assessment of mitochondrial heteroplasmy is important for diagnostics of mitochondrial dysfunction, as the clinical severity of diseases associated with mitochondrial dysfunction depends not only on the presence of mtDNA mutation but also on the proportion of mutant and normal copies of mtDNA [134]. The exceeded level of mtDNA heteroplasmy leads to impaired ATP synthesis and oxidative stress. In some tissues, heteroplasmy may be low and not cause symptoms, while in others it may reach a threshold level and lead to mitochondrial dysfunction. Numerous studies have identified an association between mtDNA mutations, such as single nucleotide polymorphisms, insertions and deletions of nucleotides, and the development of certain diseases [135,136,137]. For example, it was shown that variants of mtDNA heteroplasmy m.7681C<T and m.310insC are associated with preeclampsia [138], m.3243A>G with diabetes mellitus [139], variants m.5178C>A, m.652delG, m.12315G>A and m.3256C>T positively correlated, and m.13513G>A, m.652insG, and m.14846G>A negatively correlated with carotid atherosclerosis [10]. At the same time, molecular genetic diagnostic of mitochondrial heteroplasmy is currently based on circulating leukocyte DNA, while heteroplasmy levels in the same patient may significantly vary from one tissue to another. In particular, the study in patients with mitochondrial myopathy demonstrated that levels of mtDNA heteroplasmy m.586G>A, m.601G>A, m.616T>C changed from 5% in circulating blood leukocytes to 70% in muscle tissue [140]. Thus, the development of test systems based on the qPCR analysis of mtDNA heteroplasmy has serious limitations associated with the fact that the same mutation can be distributed differently between tissues; the level of heteroplasmy is highly variable and depends on the technical conditions during amplification and sequencing.

### 3.4. Single-Cell Sequencing

Single-cell sequencing now allows us to provide detailed, cell-type-resolved analysis of mitochondrial dysfunction at the levels of mtDNA and mRNA, revealing cell heterogeneity and subclinical pathological changes at early stages of the diseases. Single-cell mtDNA sequencing allows us to detect mtDNA mutations at the single-cell level, providing detailed insight into mitochondrial heteroplasmy in special cell types [141]. Mutations of mtDNA are significantly more common than nuclear DNA mutations. Specifically, the number of mtDNA mutations in human blood cells can be more than 200 times higher than nuclear DNA mutations. However, the presence of mtDNA mutations does not always lead to phenotypic changes, since clinical manifestations appear when the number of mutant mtDNA variants in the tissue reaches a threshold level; moreover, the threshold level of mtDNA heteroplasmy leading to mitochondrial dysfunction may vary in different cell types [142]. In recent years, single-cell sequencing studies have enabled massively parallel sequencing of the mitochondrial genome and demonstrated extremely high levels of variability in mtDNA variants and copy numbers across thousands of cells [143]. The high level of mtDNA variability between different cells, the ambiguous understanding of the phenotypic threshold for mtDNA heteroplasmy, and the technical challenges associated with amplification artifacts significantly limit the use of single-cell mtDNA sequencing in the development of diagnostic algorithms for diseases associated with mitochondrial dysfunction. At the same time, single-cell sequencing can be considered a promising method for studying pathogenetic mechanisms in oncology and developing diagnostic and therapeutic strategies based on the analysis of mtDNA and mRNA in tumor cells [144].

### 3.5. Flow Cytometry: Mitochondrial Membrane Potential and ROS Detection

Flow cytometry is a sensitive method for detecting mitochondrial dysfunction based on the determination of mitochondrial membrane potential (MMP), mitochondrial mass and mitochondrial ROS detection. Changes in mitochondrial membrane potential are directly related to mitochondrial dysfunction, since metabolic disturbances or damage to the mitochondrial membrane can lead to a decrease in MMP [145]. A decrease in MMP leads to ATP deficiency, as well as changes in pH in the intermembrane space and mitochondrial matrix. A number of mitochondrial-specific fluorescent probes have been developed for the study of MMP by flow cytometry; they can be used to measure MMP, mitochondrial mass, and the level of reactive oxygen species in mitochondria [146]. In particular, JC-1 and rhodamine 123 are used for MMP analysis [147]: JC-1 forms dimers in polarized mitochondria, giving a red signal, and monomers in depolarized mitochondria, giving a green signal; rhodamine 123 accumulates in mitochondria proportionally to MMP. Mitotrackers green, deep red, orange, and red bind to mitochondria, allowing their mass and morphology to be assessed [148], mitoSOX red is oxidized by superoxide in mitochondria, allowing the assessment of mitochondrial ROS [148]. MitoCLox is a mitochondria-targeted probe sensitive to lipid peroxidation [149]. Flow cytometry allows monitoring parameters of mitochondrial dysfunction, avoiding artifacts associated with the isolation and permeabilization of mitochondria, and has several advantages, such as working in a preserved cellular environment, using a minimal amount of biological material, and high throughput. However, this method is effective for in vitro studies, as well as for studying mitochondria in circulating peripheral blood cells [150], but its application to assess mitochondria in other tissues is significantly complicated.

### 3.6. Electron Microscopy

Electron microscopy (EM) remains the gold standard for studying mitochondrial morphology and their role in the pathogenesis of various diseases. Modern EM methods, particularly 2D and 3D microscopy, allow for a qualitative and quantitative assessment of mitochondrial ultrastructure, including cristae architecture, their phenotypes, and the contact zone with the endoplasmic reticulum and Golgi complex [151].

The classic method for assessing mitochondria is transmission electron microscopy (TEM), which, using various software, allows for the evaluation of parameters such as the total number of mitochondria, their area, perimeter, length, width, Feret’s diameter, the total number of organelles, and their volume density (the ratio of the total mitochondrial area to the total cell area) [152]. An important aspect is the ultrastructural analysis of mitochondrial cristae, which are invaginations of the inner mitochondrial membrane that house the respiratory chain and ATP synthase complexes. Normally, cells exhibit two cristae phenotypes: tubular cristae with a high base area-to-volume ratio and lamellar cristae, which are lamellar cristae that provide maximum packing density for ATP synthases and high levels of oxidative phosphorylation [153,154]. Under pathological conditions accompanied by cellular stress, cristae can transform into various shapes: arc-shaped, septa/onion, or small spherical vesicular cristae. Quantitative analysis of cristae includes determination of the number of cristae, cristae surface area (CSA), cristae volume density (CVD), as well as assessment of the crista junction diameter, intermembrane space diameter, intracristae space width, and crista junction width. Changes in these parameters are often associated with oligomerization processes of inner membrane proteins [155]. It is well known that mitochondria are involved in dynamic spatial interactions with the endoplasmic reticulum (ER), Golgi complex, lysosomes, etc. Quantitative analysis of mitochondrial contact zones with these organelles allows us to consider mitochondria as a non-isolated dynamic system that determines the rate and efficiency of fundamental biochemical processes in the cell. For example, determining the contact zone between mitochondria and the ER can indicate calcium homeostasis in the cell: if the gap widens, calcium dissipates into the cytoplasm, leading to cellular energy deficiency [156,157].

Despite its high resolution, 2D TEM analysis has several limitations for interpreting mitochondrial volumetric parameters. To understand the full three-dimensional architecture of mitochondria, volumetric electron microscopy (3D EM) is used, particularly serial block face-scanning electron microscopy (SBF-SEM) and focused ion beam-scanning electron microscopy (FIB-SEM) [151]. Three-dimensional reconstruction allows for the calculation of true volume, surface area, sphericity index, as well as the mitochondrial complexity index and branching index [158]. One of the most relevant areas of modern diagnostics is correlative light and electron microscopy (CLEM), which combines intravital fluorescence microscopy with subsequent EM fixation. This approach allows us to link the functional activity of mitochondria, in particular the membrane potential of isolated cristae, with their configuration at the ultrastructural level [159].

Mitochondrial dysfunction research has now expanded beyond the fundamental science, becoming a strategically important area for understanding the pathogenesis of a wide range of diseases. A review of modern methodological approaches demonstrates a remarkable analytical arsenal: from high-throughput transcriptomic analysis and single-cell sequencing to real-time functional testing of metabolism (Seahorse) and ultrastructural 3D modeling such as FIB-SEM, CLEM. Each method has unique diagnostic potential, but their direct integration into routine clinical practice is associated with a number of fundamental and technological challenges. Table 2 illustrates the key advantages and limitations of modern methods for assessing mitochondrial function and their potential for clinical translation.

Successful implementation of the discussed methods in clinical practice requires a shift from the isolated use of individual methods or biomarkers to a multimodal approach. Currently, the most promising direction is the validation of key mitoDEG panels using available qPCR and Western blotting methods as minimally invasive screening tests. Bioenergetic profiling of accessible cells, such as circulating monocytes, using the Seahorse assay or flow cytometry, can serve as a system indicator of the metabolic state of hard-to-reach tissues. However, the standardization of sample preparation protocols, the minimization of artifacts, and the creation of reference databases for various pathologies remain necessary to transform these methods into diagnostic standards.

## 4. Conclusions

Mitochondrial dysfunction plays a significant role in the pathogenesis of socially significant diseases, including cardiovascular pathology, neurodegenerative disorders, metabolic syndrome, and cancer, disrupting energy balance, increasing oxidative stress, and activating apoptotic pathways. Modern diagnostic methods, such as transcriptome analysis to identify the expression of genes associated with OXPHOS, apoptosis, and mitochondrial dynamics; qPCR to evaluate the level of mtDNA heteroplasmy and mtDNA copy number; single-cell sequencing to analyze the heterogeneity of cell populations; Seahorse assay to assess respiratory function; and flow cytometry to study mitochondrial membrane potential, provide a comprehensive, multi-level assessment of mitochondrial status. A combination of molecular genetic analysis and functional morphological methods reflecting the current metabolic status and ultrastructure of organelles will allow us to understand the pathogenetic mechanisms of studied diseases and to create an objective platform for early diagnosis, prognosis, and evaluation of the effectiveness of personalized therapy for diseases associated with mitochondrial dysfunction. Further development of multi-omics platforms will enhance the predictive value of diagnostics, opening up prospects for timely prevention of diseases associated with mitochondrial dysfunction and monitoring the effectiveness of mitochondrial-targeted preparations.

## Figures and Tables

**Table 1:** Differentially expressed genes associated with mitochondrial dysfunction.

Gene	Protein/Function	Results of the Studies
*MTHFD2*	mitochondrial enzyme MTHFD2 is essential for one-carbon metabolism	knockdown of *MTHFD2* leads to increased apoptosis in a murine model of intracerebral hemorrhage [88]; increased expression in glioblastoma tissues [89]; increased expression in mice with heart failure with preserved ejection fraction [90]; induction of collagen synthesis by lung fibroblast in the pathogenesis of pulmonary fibrosis [91]; stimulation of osteoclastogenesis in mice with arthritis [92]
*BCL2*	mitochondrial outer membrane protein that suppresses apoptosis	biomarker for chronic obstructive pulmonary disease [87]; regulation of apoptosis in neurodegeneration and after myocardial ischemia-reperfusion injury [93,94]
*FOXO1*	transcription factor FOXO1	regulation of mitochondrial dynamics, lipid and glucose metabolism of cardiomyocytes [95]; biomarker for chronic obstructive pulmonary disease [87]
OXPHOS genes (*COX*, *ATP5*, *NDUF*)	subunits of the mitochondrial respiratory chain complex	downregulation of *NDUF*, *COX* and *ATP5* caused by increased *S100A9* expression is associated with the development of pathological changes in the myocardium due to mitochondrial dysfunction [96,97]
*TFAM*	mtDNA binding protein and transcription factor	reduced *TFAM* expression is associated with destruction of cristae and decrease in MMP and ATP production [98]; oxidative stress stimulates mitochondrial biogenesis via increase in *TFAM* expression resulting in increase in mitochondrial number and ATP production [99]
group of genes *SLC25A*	carrier proteins involved in transport across the mitochondrial membrane	elevated in brain of mice with depression-like disorder and correlated with *Mrps* and *Mrpl* gene expression increasing due to the load on the mitochondrial respiratory chain [100]
*OPA1*	GTPase OPA1 responsible for mitochondrial fusion	increased *OPA1* expression in mice with hepatic steatosis leading to amelioration of oxidative stress due to mitochondrial dysfunction [101]
*PINK1*/ *Parkin*	mitophagy activating proteins	activation of PINK1/Parkin-dependent mitophagy leads to amelioration of fatty acid β-oxidation and mitochondrial function [102]
*Decr1*	mitochondrial enzyme Decr1 involved in the tricarboxylic acid cycle	increased expression in mice with heart failure with preserved ejection fraction [90]

ATP, adenosine triphosphate; ATP5, ATP synthetase complex V; BCL2, B-cell lymphoma 2 protein; COX, cytochrome c oxidase; FOXO1, forkhead box protein O1; mtDNA, mitochondrial DNA; MMP, mitochondrial membrane potential; MTHFD2, methylenetetrahydrofolate dehydrogenase 2; NDUF, NADH: ubiquinone oxidoreductase flavoprotein; OXPHOS, oxidative phosphorylation; SLC25A, solute carrier family 25 member; TFAM, mitochondrial transcription factor A.

**Table 2:** The advantages and limitations of modern methods for assessment of the mitochondrial function.

Methods	Advantages	Limitations	Current Clinical Readiness
Transcriptome analysis	• identification of specific mitoDEGs for a wide range of socially significant diseases; • allows assessing the changes in gene expression before clinical manifestations of pathology.	• does not reflect the actual metabolic activity of the encoded proteins; • requires validation by other methods for correct assessment of mitochondrial functions.	Research-grade. Limited clinical use. Not standardized for mitochondrial diagnostics; gene panels undergoing validation.
Seahorse extracellular flux assay	• highly sensitive; • allows real-time assessment of key metabolic parameters such as glycolysis, mitochondrial respiration and fatty acid oxidation; • allows providing stress tests in isolated organelles or living cells.	• high data variability due to technical conditions; • in vitro analysis does not reproduce intercellular interactions and tissue specifics; • limited in duration, does not allow recording long-term effects without the use of additional data such as proteomics or mtDNA analysis.	Purely research. Not used in routine diagnostics due to technical complexity and requirement for living cells.
qPCR	• determination of the mtDNA copy number used as a biomarker of several chronic diseases; • identification of point mtDNA mutations, insertions and deletions; • assessment of the mtDNA heteroplasmy threshold determining the clinical severity of the pathology.	• diagnostics is most often based on DNA of blood leukocytes, whereas the level of heteroplasmy varies greatly between tissues; • results are highly variable and depend on the technical conditions of amplification.	Clinically applicable. Used in specialized centers for inherited mitochondrial diseases, but limited by tissue specificity and standardization issues.
Single-cell sequencing	• provides detailed analysis at the level of individual cells types (resolution of cellular heterogeneity); • effective in studying oncogenesis (analysis of mtDNA/mRNA of tumor cells).	• technical difficulties associated with amplification artifacts; • high variability of mtDNA copy number; • the absence of criteria for the phenotypic threshold of mtDNA heteroplasmy for individual cell types.	Experimental/research only. Not clinically implemented due to cost and lack of criteria.
Flow cytometry	• evaluates parameters in a native cellular microenvironment without organelle isolation artifacts; • requires a minimal amount of biomaterial; • comprehensive measurement of MMP, mitochondrial mass, ROS, and lipid peroxidation.	• effective primarily for in vitro research or for the analysis of peripheral blood cells; • isolation and evaluation of mitochondria from dense parenchymal tissues is significantly complicated.	Translational. Used in clinical labs, but mitochondrial parameters not yet standardized. Promising for blood-based monitoring.
Electron microscopy	• the “gold standard” for nanoscale analysis of mitochondrial geometry and cristae parameters; • 3D EM (FIB-SEM) and CLEM methods allow the reconstruction of the true network volume and the correlation of morphology with the intravital membrane potential.	• fixation precludes the observation of dynamics (fission/fusion) in real time; • low availability and labor-intensive sample preparation.	Clinically used (limited). Gold standard for morphology in specialized pathology centers, but not routine.

## Data Availability

No new data were created or analyzed in this study.
